# Preparation and Application of a Magnetic Oxidized Micro/Mesoporous Carbon with Efficient Adsorption for Cu(II) and Pb(II)

**DOI:** 10.3390/polym14224888

**Published:** 2022-11-12

**Authors:** Jia Qu, Hongpu Huang, Qiang Yang, Wei Gong, Meilan Li, Liangliang Chang, Baoyue Cao, Guochun Zhang, Chunsheng Zhou

**Affiliations:** 1 Shaanxi Key Laboratory of Comprehensive Utilization of Tailings Resources, Shaanxi Engineering Research Center for Mineral Resources Clean & Efficient Conversion and New Materials, Shangluo University, Shangluo 726000, China; 2 School of Materials Science and Engineering, Yangtze Normal University, Chongqing 408100, China

**Keywords:** magnetic mesoporous carbon, adsorption, copper, lead, adsorption kinetics, adsorption isotherms, selectivity

## Abstract

Water pollution is a worldwide problem that requires urgent attention and prevention and exceeding use of heavy-metal ions is one of the most harmful factors, which poses a serious threat to human health and the ecological environment. In this work, a magnetic oxidized micro/mesoporous carbon (MOMMC) was prepared for the easy separation of Cu(II) and Pb(II) from water. The dual-template method was used to prepare micro/mesoporous carbon using sucrose as the carbon source, silica nanoparticles formed by tetraethyl orthosilicate as the microporous templates, and triblock copolymer F127 as the mesoporous template. MOMMC was obtained by oxidation using potassium persulfate and then magnetized through in situ synthesis of Fe_3_O_4_ nanoparticles. FTIR, TG-DSC, XRD, TEM, SEM, nitrogen adsorption–desorption isotherms, zeta potential, and VSM were used to confirm the synthetic process, structure, and basic properties of MOMMC. The high-saturation magnetization (59.6 emu·g^−1^) of MOMMC indicated its easy and fast separation from water by an external magnetic field. Kinetics studies showed that the adsorption of Cu(II) and Pb(II) on MOMMC fit the pseudo-second-order model well. Isotherm studies showed that the adsorption behavior of Cu(II) was better described by the Langmuir model, and the adsorption behavior of Pb(II) was better described by both Langmuir and Redlich–Peterson models. MOMMC obtained efficient adsorption for Cu(II) and Pb(II) with the large adsorption capacity of 877.19 and 943.40 mg·g^−1^ according to the Langmuir adsorption isotherm equation, and a better selectivity for Pb(II) was observed in competitive adsorption. MOMMC still possessed a large adsorption capacity for Cu(II) and Pb(II) after three adsorption–desorption cycles. These findings show that MOMMC represents an excellent adsorption material for the efficient removal of heavy-metal ions.

## 1. Introduction

Water pollution is a worldwide problem that requires urgent attention and prevention and exceeding use of heavy-metal ions is one of the most harmful factors, which poses a serious threat to human health and the ecological environment [1,2]. Among the various heavy metals, copper is reported as a widespread one in wastewater, which may come from natural or anthropogenic sources, such as mining activities, fertilizers, pesticides, paints, etc. [3]. Although copper is an essential micronutrient, it can cause human poisoning at high concentrations [4]. Lead has also been studied in water treatment due to its high toxicity to human health even at low concentration [5]. The main origins of lead pollution are automobiles, paint, and electroplating industries [6]. Methods for the removal of heavy metals include chemical precipitation, chemical oxidation or reduction, electrochemical treatment, evaporative recovery, filtration, ion exchange, membrane technologies, and adsorption [7]. Among the various water purification and recycling technologies, adsorption is deemed to be an effective, practicable, and economic method [8,9].

For water treatment, large adsorption capacity, fast adsorption speed, high mechanical strength, high stability, and low cost are the basic requirements of adsorbents [10,11]. Mesoporous carbon material refers to a kind of porous carbon with a pore size between 2 and 50 nm, which has a high specific surface area and can provide a large number of active sites for adsorption. Generally, the adsorption capacity of mesoporous carbon is correlated with its specific surface area, and the existence of micropores is conducive to improving the specific surface area of the material [12,13]. Carbon materials with mesopores as well as micropores can be constructed by the interaction between the double template and the carbon precursor [14]. As for the adsorption speed, it is mainly related to the particle size and pore size distribution of the adsorbent. Mesoporous carbon materials have suitable pore size distribution, and the abundant mesopore is conducive to the diffusion of adsorbate in pores [15]. In addition, mesoporous carbon material is of high mechanical strength, chemically stable, thermostable, non-toxic, and durable, so it is considered an excellent adsorption material [16]. It is noteworthy that the surface of mesoporous carbon is easily modified. As a heavy-metal ion adsorbent, the modification of carboxyl, amino, and other functional groups on the surface of mesoporous carbon can not only improve the hydrophilicity of carbon materials, but also produce complexation or electrostatic attraction with heavy-metal ions, which can effectively improve the adsorption capacity and selectivity of mesoporous carbon materials [17,18]. Although mesoporous carbon and its modified materials have excellent adsorption properties, the separation of mesoporous carbon from water is difficult. If this problem cannot be solved properly, the application of mesoporous carbon in the field of water treatment will have the possibility of secondary pollution. Magnetic separation technology, in comparison with other methods such as centrifugation, precipitation, and filtration, has exhibited easy operation and reduced capital costs [19]. Therefore, magnetic separation is an attractive option because it allows easy recovery of the adsorbent by an external magnetic field.

Based on this view, we prepared a magnetic oxidized micro/mesoporous carbon (MOMMC) material for the adsorption of Cu(II) and Pb(II). Sucrose was used as the carbon source, silica nanoparticles formed by tetraethyl orthosilicate (TEOS) were used as microporous templates, and the commercial triblock copolymer F127 was used as the mesoporous template. Mesoporous carbon-silica nanocomposites had been synthesized by the evaporation-induced tri-constituent co-assembly method, followed by calcination [14]. Then, the micro/mesoporous carbon (MMC) material was obtained by NaOH etching. Finally, MOMMC material was obtained by oxidation using potassium persulfate and then magnetized through in situ synthesis of Fe_3_O_4_ nanoparticles in the pore and surface of oxidized micro/mesoporous carbon. FTIR, TG-DSC, XRD, TEM, SEM, nitrogen adsorption–desorption isotherms, zeta potential, and VSM were used to confirm the synthetic process, structure, and basic properties of MOMMC. It should be noticed that modeling and calculation are of great value in adsorption studies [20,21,22,23,24], so we used a large number of adsorption kinetics models and adsorption isotherm models to better understand the adsorption behavior of Cu(II) or Pb(II) on MOMMC. The adsorption capacity of Cu(II) or Pb(II) on MMC and MOMMC were compared, and the recovery performance and competitive adsorption were also considered.

## 2. Materials and Methods

### 2.1. Materials

Poly(ethylene glycol)-block-poly(propylene glycol)-block-poly(ethylene glycol) triblock copolymer (Pluronic F127, *M*_w_ = 14,600), tetraethyl orthosilicate (TEOS, 98%), sucrose, sodium hydroxide (NaOH, 97%), potassium persulfate (K_2_S_2_O_8_, 99.5%), potassium hydroxide (KOH, 95%), iron(III) chloride hexahydrate (FeCl_3_·6H_2_O, 99%), iron(II) chloride tetrahydrate (FeCl_2_·4H_2_O, 99%), ammonia solution (NH_3_·H_2_O, 25–28%), and ethylenediaminetetraacetic acid disodium salt dihydrate (EDTA-2Na, 98%) were purchased from Aladdin (Shanghai, China). Hydrochloric acid (HCl, 36%), ethanol (99.7%), copper(II) sulfate pentahydrate (CuSO_4_·5H_2_O, 99%), and lead(II) nitrate (Pb(NO_3_)_2_, 99%) were purchased from Chengdu Kelong Chemical Co., Ltd. (Chengdu, China). All chemicals were used as-received without any further purification.

### 2.2. Preparation of Micro/Mesoporous Carbon

**Scheme 1:**
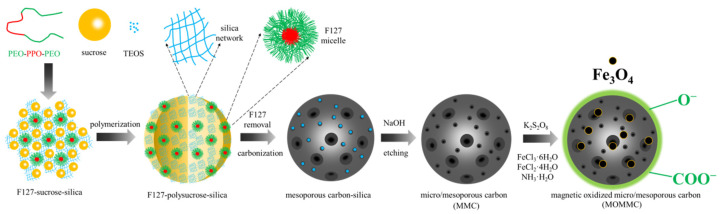
Synthesis of MMC and MOMMC.

Here, 1.8 g of F127 and 1 mL of hydrochloric acid were mixed in 10 mL of ethanol, and the mixed solution was stirred at 50 °C for 1 h. A certain amount of sucrose and TEOS were added to the above solution, and the reaction was carried out with stirring for 2 h. The product was evenly coated on the dishes, evaporated for 8 h at room temperature, and then placed in an oven at 120 °C for 24 h to obtain the film. Sucrose had been polymerized and TEOS had been translated into silica via the sol-gel method, and the obtained dark amber film was denoted as F127-polysucrose-silica. Calcination was carried out for F127-polysucrose-silica in a tubular furnace at 350 °C for 3 h and then 850 °C for 2 h under nitrogen flow to obtain carbon-silica. The heating rate was 3 °C·min^−1^ during the whole heating process. After calcination, the carbon-silica nanocomposite was put into a flask containing 5 mol·L^−1^ of NaOH and refluxed at 50 °C for 24 h to remove the silica. Finally, the micro/mesoporous carbon material (denoted as MMC) was obtained. The synthesis scheme of MMC is presented in Scheme 1, and the detailed recipes are listed in Table 1.

### 2.3. Preparation of Magnetic Oxidized Micro/Mesoporous Carbon

Magnetic oxidized micro/mesoporous carbon was synthesized in two steps. First, micro/mesoporous carbon was oxidized by potassium persulfate. Then, 0.4 g micro/mesoporous carbon was dissolved in 600 mL of deionized water followed by the addition of 18 g of potassium persulfate, and the pH of the reaction system was adjusted to 12 using 1 mol·L^−1^ of KOH. The oxidation reaction was conducted through continuous stirring at 80 °C for 3 h. Oxidized micro/mesoporous carbon was finally obtained after centrifugation, rinsing with deionized water and vacuum-drying at 50 °C. Second, oxidized micro/mesoporous carbon was magnetized through in situ synthesis of Fe_3_O_4_. Here, 0.581 g of FeCl_3_·6H_2_O and 0.375 g of FeCl_2_·4H_2_O were dissolved in 37.5 mL of deionized water in a flask, followed by raising the temperature to 70 °C under mechanical agitation accompanied by nitrogen protection. After adding 12.5 mL of ammonia solution, 0.25 g of oxidized micro/mesoporous carbon was quickly added into the flask and the reaction was carried out at 70 °C for 1 h and then 85 °C for another 1 h. Magnetic oxidized micro/mesoporous carbon (denoted as MOMMC) was finally obtained after centrifugation, rinsing with deionized water and vacuum-drying at 50 °C. The synthesis scheme of MOMMC is also presented in Scheme 1.

### 2.4. Characterization of MMC and MOMMC

**Scheme 2:**
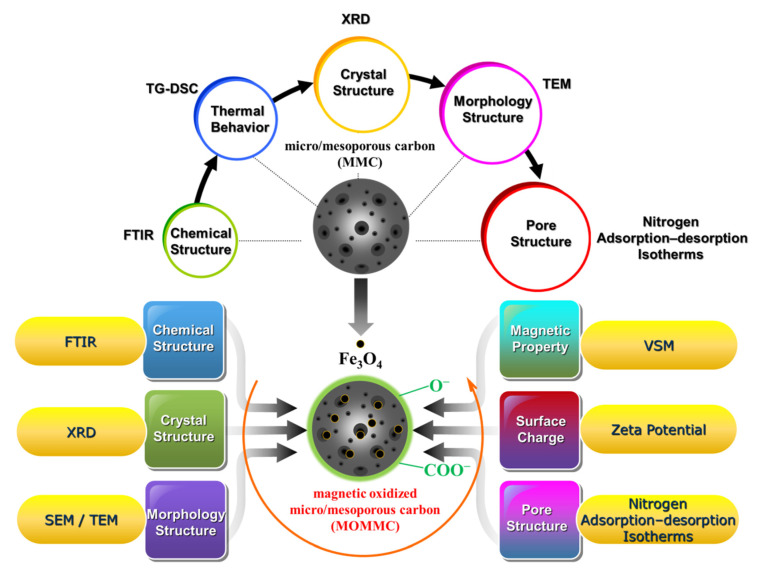
Characterization of MMC and MOMMC.

A Fourier transform infrared spectrometer (FTIR, Nicolet-380, Thermo Electron Corporation, Waltham, MA, USA) was used to verify the chemical structure of MMC and MOMMC in 4000–400 cm^−1^ using the potassium bromide pellet technique. A thermogravimetric analyzer (TG-DSC, STA449, Netzsch, Germany) was used to study the process of MMC formation under N_2_ atmosphere using a heating rate of 10 °C·min^−1^ from 25 to 850 °C. An X-ray diffractometer (XRD, X’Pert Powder, Panalytical, Netherlands) with Ni-filtered Cu Kα radiation (40 kV, 40 mA) was used to understand the crystal structure of MMC and MOMMC in a 2 theta of 15–80°. Transmission electron microscopy (TEM, Talos F200X, FEI, Czech Republic) was used to observe the morphologies of MMC and MOMMC at an acceleration voltage of 200 kV. Scanning electron microscopy (SEM, Sigma 300, Zeiss, Germany) was used to further observe the existence of Fe_3_O_4_ on MOMMC. A specific surface area and porosity analyzer (ASAP 2460, Micromeritcs, Norcross, GA, USA) was used to measure nitrogen adsorption–desorption isotherms of MMC and MOMMC at 77 K for the study of the pore structure. The surface area was determined based on the Brunauer–Emmett–Teller (BET) method. Mesoporous size distribution was derived from the desorption branches based on the Barrett–Joyner–Halenda (BJH) model. Micropore size distribution was derived from the desorption branches based on the Horvath–Kawazoe (HK) model. Zeta potential was measured by dispersing MOMMC in deionized water by sonication, and then determining the suspension with the Zetasizer Nano (ZEN3600, Malvern, UK). The magnetic property of MOMMC was studied by a vibrating sample magnetometer (VSM, 7404, LakeShore, Columbus, Ohio, USA) at 25 °C. The characterization scheme of MMC and MOMMC is presented in Scheme 2.

### 2.5. Batch Adsorption Study

Here, 0.02 g of MMC or MOMMC was added into 20 mL of Cu(II) or Pb(II) aqueous solutions on a shaker at 120 rpm under 25 °C and a certain pH value (pH = 2, 4, 5, 6, 8, 10). After shaking for a certain period of time, supernatant separated from MMC by centrifugation at 13,500 rpm or separated from MOMMC by magnetism using a magnet was analyzed to detect the residual concentration of Cu(II) or Pb(II). The concentration of residue Cu(II) or Pb(II) was determined using an atomic absorption spectrometer (AAS, AA-7003, EWAI, China).

The equilibrium absorption capacity, *Q*_e_ (mg·g^−1^), was calculated according to Equation (1) [25]. The removal efficiency, *η* (%), was calculated according to Equation (2) [26]:

*Q* = (*C*_0_ − *C*_t_)*V*/*W* (1)

*η* = 100(*C*_0_ − *C*_t_)/*C*_0_ (2)

where *C*_0_ (mg·L^−1^) and *C*_t_ (mg·L^−1^) are the initial concentrations and equilibrium concentrations of Cu(II) or Pb(II), *V* (L) is the volume of the solution, and *W* (g) is the weight of the adsorbent.

### 2.6. Adsorption Kinetics

Adsorption kinetics were carried out under an initial Cu(II) or Pb(II) concentration of 1000 mg·L^−1^, initial pH of 5, and a controlled time, *t* (5, 10, 20, 30, 60, 90, 120, 180, and 240 min). The pseudo-first-order model expressed by Equation (3) [25] and pseudo-second-order model expressed by Equation (4) [25] were used to analyze the adsorption rate:

ln(*Q*_e_ − *Q*_t_) = −*k*_1_*t* + ln*Q*_e_ (3)

*t*/*Q*_t_ = *t*/*Q*_e_ + 1/(*k*_2_*Q*_e_^2^) (4)

where *Q*_e_ (mg·g^−1^) and *Q*_t_ (mg·g^−1^) are the equilibrium adsorption capacity and the adsorption capacity at time *t* (min), and *k*_1_ (min^−1^) and *k*_2_ (g·mg^−1^·min^−1^) are the rate constants.

### 2.7. Adsorption Isotherms

Adsorption isotherms were measured by controlling the Cu(II) or Pb(II) concentration at 50, 100, 200, 500, and 1000 mg·L^−1^, respectively. After 120 min of equilibrium at an initial pH of 5, the supernatant was measured for the concentration of residual Cu(II) or Pb(II).

The Langmuir, Freundlich, Dubinin–Radushkevich, Temkin, and Redlich–Peterson models were investigated according to Equation (5) [25], Equation (6) [25], Equation (7) [25], Equation (8) [25], and Equation (9) [27], respectively. The goodness-of-fit was determined by the correlation coefficient (*R*^2^) and the average relative error (ARE) calculated from Equation (10) [27].

*C*_e_/*Q*_e_ = 1/(*Q*_m_*K*_L_) + *C*_e_/*Q*_m_ (5)

ln*Q*_e_ = ln*K*_f_ + (1/*n*)ln*C*_e_ (6)

ln*Q*_e_ = ln*Q*_m_ − *βε*^2^ (7)

*Q*_e_ = (*RT*/*b*_T_)ln*A*_T_ + (*RT*/*b*_T_)ln*C*_e_ (8)

ln(*K*_R_*C*_e_/*Q*_e_ − 1) = *g*ln*C*_e_ + ln*B* (9)

(10) $$\mathrm{ARE}=\frac{100}{N}\sum_{i=1}^{N}\left|\frac{(Q_{e,exp}-Q_{e,cal}}{Q_{e,exp}}\right|$$

The meanings of each physical quantity are shown in Table 2.

### 2.8. Recovery Experiments

Adsorption–desorption cycles were carried out to evaluate the recovery performance of MOMMC. The adsorption of Cu(II) or Pb(II) was conducted at an initial concentration of 1000 mg·L^−1^, a controlled pH of 5, and a controlled temperature of 25 °C. After adsorption equilibrium, the adsorbed Cu(II) or Pb(II) was removed from MOMMC using 0.1 mol·L^−1^ of ethylenediaminetetraacetic acid disodium salt (EDTA-2Na) solution with a contact time of 24 h and a solid/liquid ratio of 0.2 g·L^−1^. MOMMC was magnetically separated, washed with deionized water, and vacuum-dried for the successive adsorption–desorption cycles.

### 2.9. Competitive Adsorption

Competitive adsorption was carried out in batch mode under an initial pH of 5 and adequate contact time of 120 min, and the initial concentrations of Cu(II) and Pb(II) were both 1000 mg·L^−1^. AAS was used to determine the residual concentration of Cu(II) or Pb(II), and the equilibrium absorption capacity, *Q*_e_ (mg·g^−1^), was calculated according to Equation (1) [25].

## 3. Results and Discussion

### 3.1. Adsorbent Characterizations

**Figure 1:**
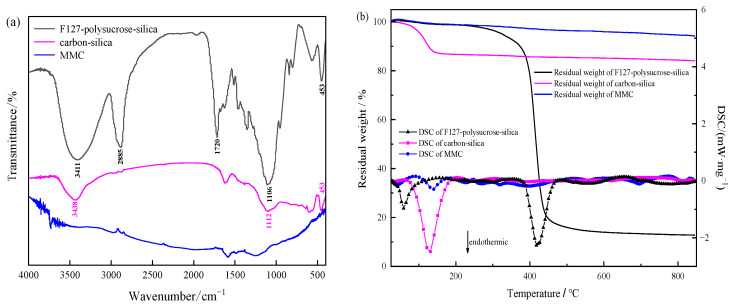
FTIR spectrum (**a**) and TG-DSC curves (**b**) of F127-polysucrose-silica, carbon-silica, and MMC.

The FTIR spectra of the samples obtained at different experimental stages during the preparation of micro/mesoporous carbon are shown in Figure 1a. As shown by the black spectrum, the O-H stretching vibration absorption peak around 3411 cm^−1^ in the uncalcined F127-polysucrose-silica was mainly attributed to the large amount of hydroxyl group in polysucrose. The C-H stretching vibration absorption peak around 2885 cm^−1^ was mainly attributed to the methyl, methylene, and methine groups in F127 and polysucrose. The absorption peak around 1720 cm^−1^ was attributed to C=O stretching vibration of polysucrose. The absorption peak around 1106 cm^−1^ was attributed to the superposition of Si-O-Si and C-O stretching vibration. The absorption peak around 453 cm^−1^ was attributed to the Si-O-Si stretching vibration of silica. After carbonization at 850 °C (carbon-silica in magenta spectrum), the C-H stretching vibration absorption peak around 2885 cm^−1^ and the C=O stretching vibration absorption peak around 1720 cm^−1^ disappeared, indicating that carbonization occurred in polysucrose, and the template F127 was completely removed. The absorption peak around 3438 cm^−1^ was attributed to the SiO-H stretching vibration of silica. The absorption peaks around 1112 and 453 cm^−1^ were attributed to the Si-O-Si stretching vibration of silica. After NaOH treatment (MMC in blue spectrum), the absorption peaks of SiO-H and Si-O-Si disappeared, indicating that silica was completely etched by NaOH.

The TG and DSC curves of F127-polysucrose-silica, carbon-silica, and MMC are shown in Figure 1b. F127-polysucrose-silica showed a sharp endothermic peak as well as a significant weight loss at 380–430 °C, which was mainly attributed to the thermal decomposition of template F127 [14]. Carbon-silica showed a sharp endothermic peak as well as a mass loss about 12 wt.% below 130 °C, which was speculated to be caused by dehydration of the hydrophilic silica obtained by the sol-gel method using TEOS as a raw material. After etching by NaOH, MMC obtained a high thermal stability, with 94.2% carbon residue at 850 °C.

**Figure 2:**
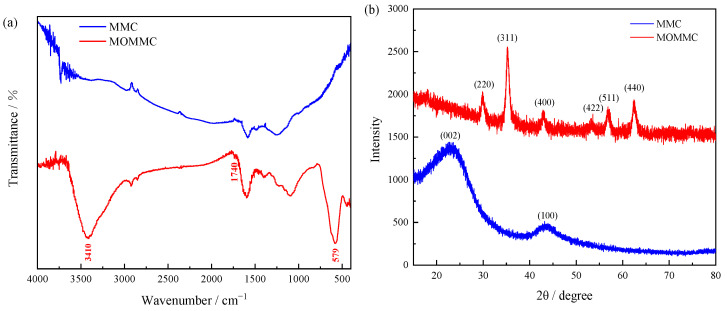
FTIR spectrum (**a**) and XRD patterns (**b**) of MMC and MOMMC.

The FTIR spectra of MMC and MOMMC are shown in Figure 2a. Compared with MMC, the O-H stretching vibration absorption peak around 3410 cm^−1^ and the C=O stretching vibration absorption peak around 1700 cm^−1^ indicated that the oxygen-containing functional groups were introduced on the surface of MOMMC, which would provide more active sites for the adsorption of heavy-metal ions. The new peak in the FTIR spectrum of MOMMC at 579 cm^−1^ was attributed to the Fe-O stretching vibration, which proved that the magnetic Fe_3_O_4_ had been successfully embedded in MOMMC [28].

The XRD patterns of MMC and MOMMC are shown in Figure 2b. The MMC showed an amorphous structure with two broad peaks around 23° and 44° in the (002) and (100) planes [14,29]. The peaks of (220), (311), (400), (422), (511), and (440) located at 29.9°, 35.3°, 43.0°, 53.6°, 56.9°, and 62.4° were the characteristic peaks of Fe_3_O_4_, which further validated that Fe_3_O_4_ nanoparticles were coated onto surfaces of carbon [28,30].

**Figure 3:**
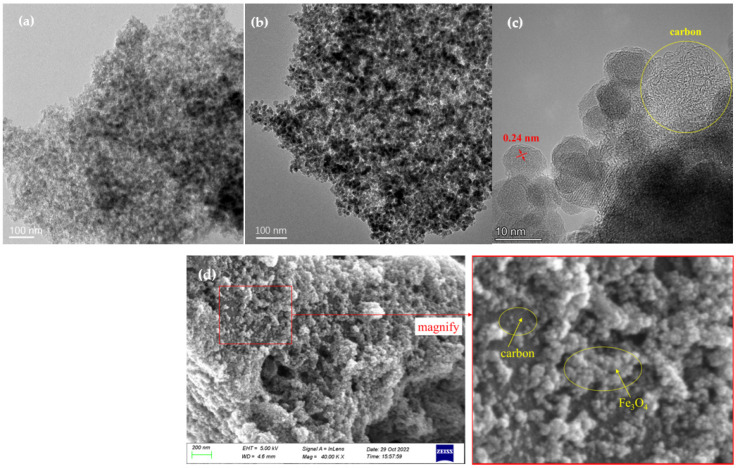
TEM image of MMC (**a**), TEM image of MOMMC (**b**), HRTEM image of MOMMC (**c**), and SEM image of MOMMC with red region magnified (**d**).

The TEM and SEM images of MMC and MOMMC are shown in Figure 3. The MMC showed a well-developed pore structure (Figure 3a), which would provide abundant active sites for the adsorption of heavy-metal ions. After functionalization, the black spherical Fe_3_O_4_ nanoparticles were found embedded in the pores and surface of porous carbon (Figure 3b). The size of the Fe_3_O_4_ nanoparticle was measured to be 8–10 nm according to the high-resolution transmission electron microscope (HRTEM) image (Figure 3c). The lattice spacing was measured to be 0.24 nm, which was assigned to the (311) plane of Fe_3_O_4_ [31]. The amorphous structure of carbon was also observed by the HRTEM image, which was consistent with the results of XRD. SEM demonstrated the morphology of MOMMC as well (Figure 3d). The gray surface was carbon, and the small white particles are the Fe_3_O_4_.

**Figure 4:**
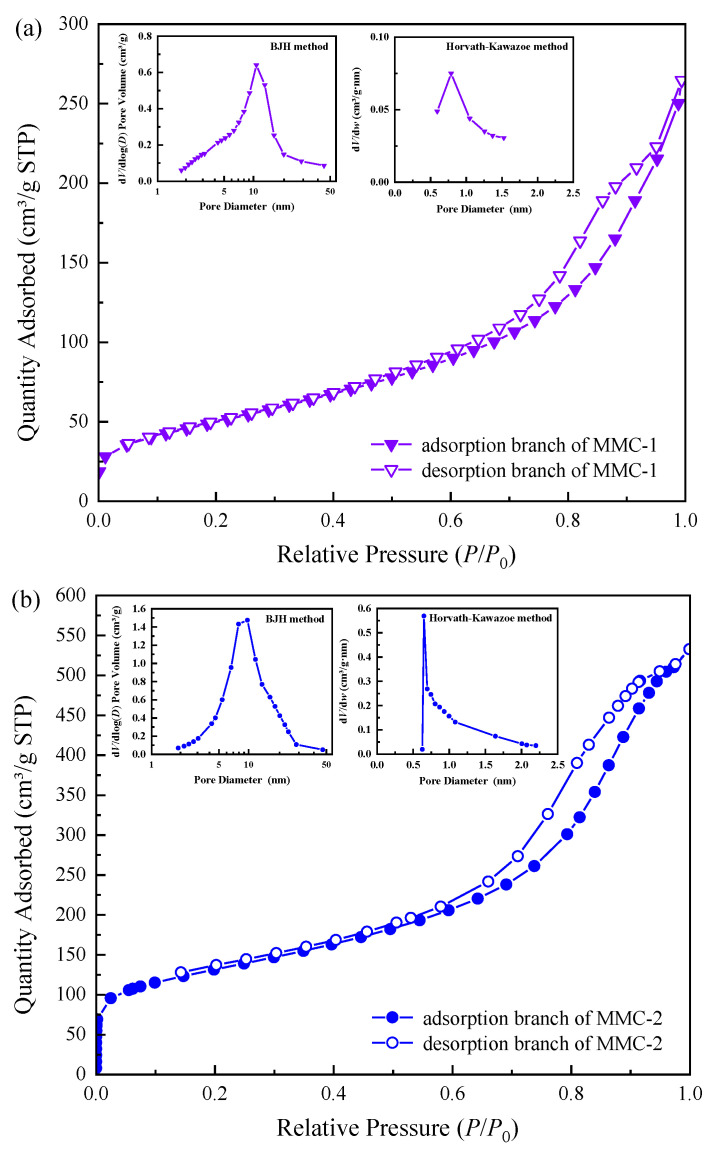

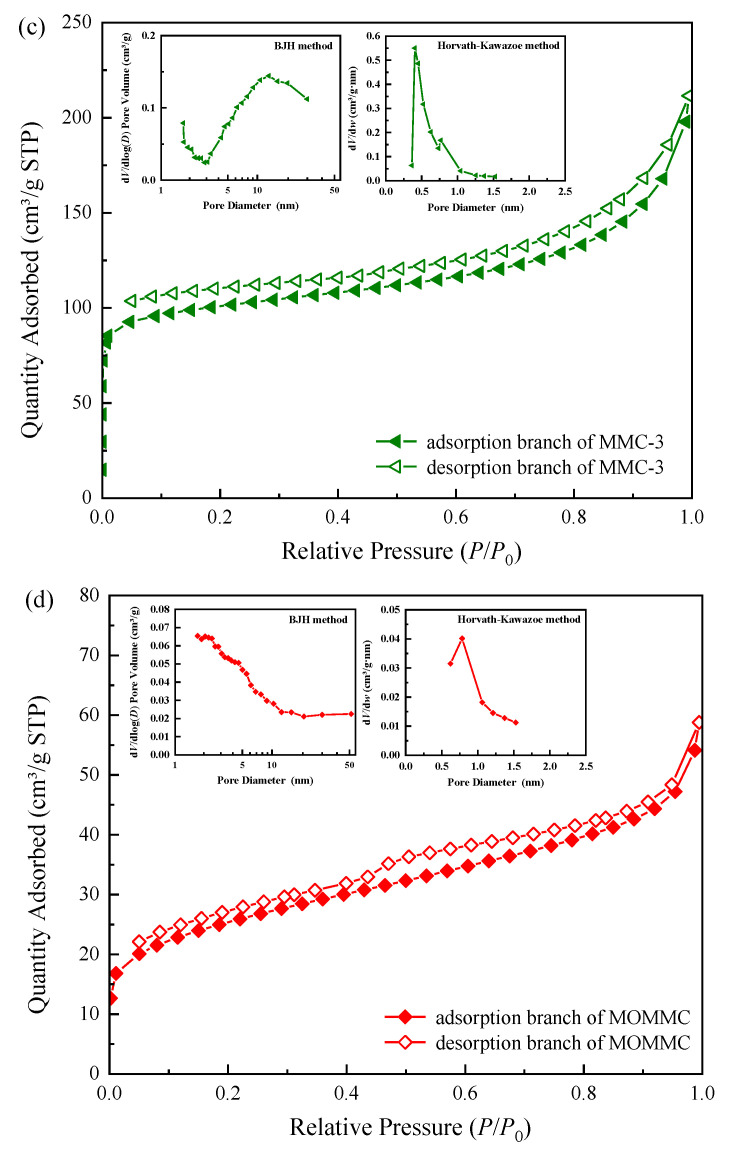
Nitrogen adsorption–desorption isotherms and the corresponding pore size distributions of MMC-1 (**a**), MMC-2 (**b**), MMC-3 (**c**), and MOMMC (**d**).

Nitrogen adsorption–desorption isotherms and the corresponding pore size distributions of MMC-1, MMC-2, MMC-3, and MOMMC are shown in Figure 4. The isotherm of MMC-1 could be classified as type IV, according to the IUPAC classification. It exhibited an H2(b) hysteresis loop, which meant a slightly wider pore distribution. The MMC-1 sample exhibited a specific surface area of 183.53 m^2^·g^−1^ and a pore volume of 0.41 cm^3^·g^−1^. The smaller surface area might be due to the small amount of micropores in the sample, which was generally an unfavorable factor for adsorption. With increased carbon sources and silica templates for micropores, the sample MMC-2 also displayed a type IV N_2_ sorption isotherm with an H2(b) hysteresis loop. Generally, with the increase of carbon sources, the specific surface area and pore volume decreased, and the pore wall thickened [32]. However, the existence of micropores was more conducive to improving the specific surface area of mesoporous materials [12,13]. Thus, the sample MMC-2 exhibited a larger specific surface area of 460.21 m^2^·g^−1^ and a higher pore volume of 0.82 cm^3^·g^−1^, which was more conducive to adsorption. With further increased carbon sources and silica templates for micropores, the sample MMC-3 displayed a type I in consort with a type IV N_2_ sorption isotherm, with an unclosed H4 hysteresis loop, indicating a smaller surface area (381.72 m^2^·g^−1^) but lots of micropores (290.12 m^2^·g^−1^). After oxidation by potassium persulfate and magnetization by Fe_3_O_4_, the sample MOMMC derived from MMC-2 showed a small surface area (89.40 m^2^·g^−1^) and pore volume (0.09 cm^3^·g^−1^). This was mainly due to the embedding of Fe_3_O_4_ nanoparticles, which leads to the blockage and occupancy of micro/mesoporous channels. However, the sample MOMMC still maintained the type IV N_2_ sorption isotherm with an H2(b) hysteresis loop, showing that it still had a definite mesoporous structure. Pore structure parameters of MMC-1, MMC-2, MMC-3, and MOMMC are summarized in Table 3.

**Figure 5:**
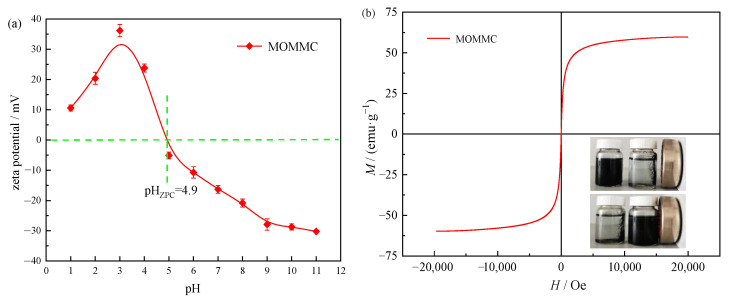
Zeta potential (**a**) and VSM magnetization curve (**b**) of MOMMC.

The zero-point charge (ZPC) of MOMMC was measured to be about 4.9, as shown in Figure 5a, indicating that the pH of the medium was an important parameter for adsorption onto MOMMC. When pH < pH_ZPC_, the surface of MOMMC would be positively charged, which was unfavorable for the adsorption of cations. When pH > pH_ZPC_, the surface of MOMMC would be negatively charged, which was favorable for the adsorption of metal cations. The saturation magnetization of the MOMMC at room temperature shown in Figure 5b was 59.6 emu·g^−1^, indicating that MOMMC possessed high magnetization and was easily separated from water through an external magnetic field. This effectively solved the problem of separating porous carbon from water, and greatly improved the practicability of the porous carbon adsorbent.

### 3.2. Adsorption Capacity of Different Adsorbents

**Figure 6:**
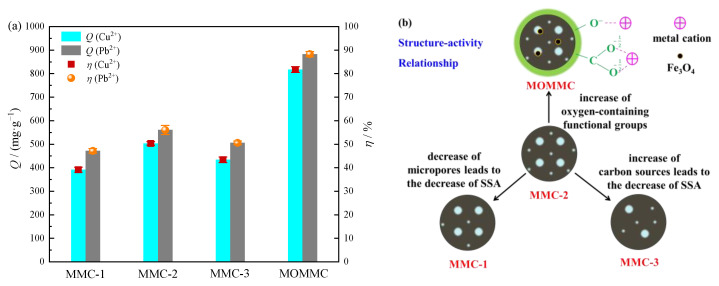
Adsorption capacity of different adsorbents (**a**) and the graphical analysis of the structure–activity relationship (**b**) (*C*_0_ = 1000 mg·L^−1^, pH = 5, *T* = 25 °C, adsorbent concentration = 1 g·L^−1^, contact time = 120 min).

The adsorption effects of different adsorbents on 1000 mg·L^−1^ Cu(II) and Pb(II) are shown in Figure 6a. The experiment was carried out for 120 min to ensure that adsorption equilibrium was reached. It could be seen that the micro/mesoporous carbon materials had an excellent adsorption effect on heavy-metal cations Cu(II) and Pb(II). The adsorption capacity of MMC-2 for Cu(II) and Pb(II) reached 503.49 and 560.54 mg·g^−1^, respectively. The better adsorption performance of MMC-2 compared with MMC-1 and MMC-3 was due to the larger specific surface area, which could provide more active sites for adsorption. After oxidation by potassium persulfate and magnetization by Fe_3_O_4_, the sample MOMMC derived from MMC-2 showed significantly higher adsorption capacity and removal efficiency. The adsorption capacity of MOMMC for Cu(II) and Pb(II) reached 817.29 and 882.87 mg·g^−1^, respectively. The removal efficiency of MOMMC for Cu(II) and Pb(II) reached 81.73% and 88.29%, respectively. MOMMC derived from MMC-2 showed a much smaller surface area (89.40 m^2^·g^−1^) and pore volume (0.09 cm^3^·g^−1^), but a much higher adsorption capacity for Cu(II) and Pb(II). This indicated that the oxygen-containing functional groups on the surface of MOMMC played an important role in the adsorption of heavy-metal cations. A covalent bond was thought to be formed between oxygen-containing functional groups and metal cations by complexation and chelation as well as electrostatic attraction, which have been confirmed by existing literature [33]. The structure–activity relationship between the adsorbents and heavy-metal cations is shown in Figure 6b.

### 3.3. Effect of pH

**Figure 7:**
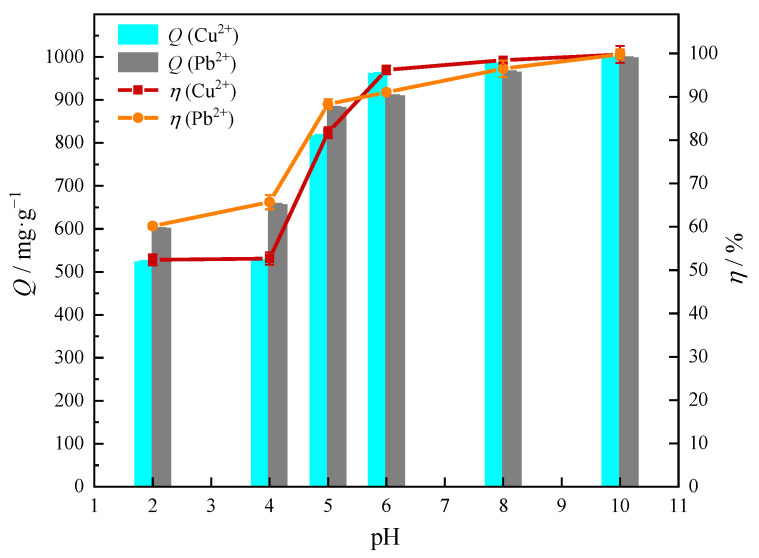
Effect of pH on adsorption capacity and removal efficiency (*C*_0_ = 1000 mg·L^−1^, *T* = 25 °C, adsorbent concentration = 1 g·L^−1^, contact time = 120 min).

The effect of pH on the adsorption of Cu(II) or Pb(II) was investigated, and the results are shown in Figure 7. As can be seen from Figure 7, the adsorption capacity of MOMMC for heavy-metal ions increased significantly with the increase of pH. This was because when pH < pH_ZPC_ = 4.9, the surface of MOMMC was positively charged and had poor adsorption performance for metal cations. When pH > pH_ZPC_ = 4.9, the surface of MOMMC was negatively charged, and the adsorption performance for metal cations was significantly improved. According to the calculation of the solubility product constant (*K*_sp_), it could be seen that when pH was greater than 5.07, Cu(II) would start to precipitate in the form of Cu(OH)_2_. When pH was higher than 7.74, Pb(II) started to precipitate in the form of Pb(OH)_2_. Therefore, pH = 5 was determined as the adsorption pH value throughout our subsequent experiments, where the adsorption capacity and removal efficiency for Cu(II) and Pb(II) were 817.29 mg·g^−1^, 81.73%, and 882.87 mg·g^−1^, 88.29%, respectively.

### 3.4. Adsorption Kinetics of MOMMC

**Figure 8:**
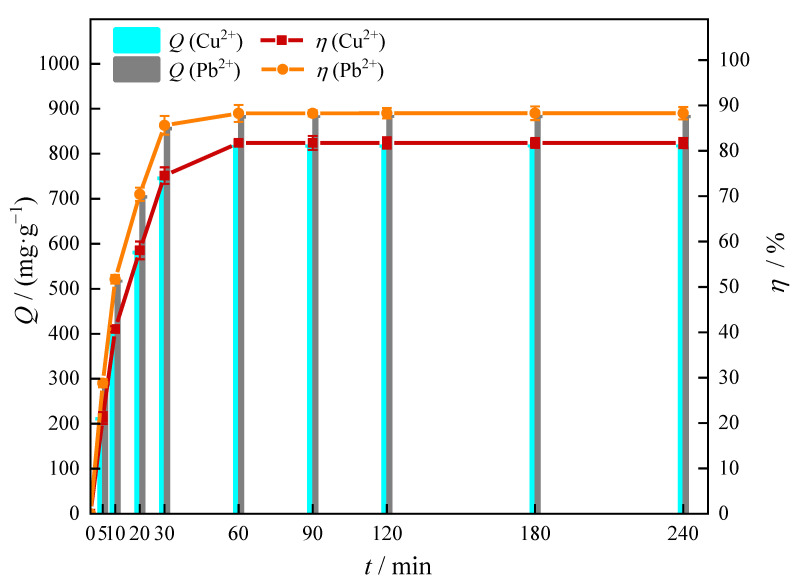
Adsorption kinetics of Cu(II) and Pb(II) on MOMMC (*C*_0_ = 1000 mg·L^−1^, pH = 5, *T* = 25 °C, adsorbent concentration = 1 g·L^−1^).

**Figure 9:**
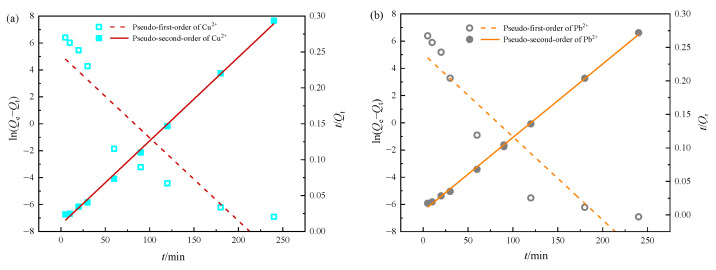
Fitting curves of adsorption kinetics for Cu(II) (**a**) and Pb(II) (**b**) on MOMMC.

Adsorption kinetics curves are shown in Figure 8. It could be seen from Figure 8 that Cu(II) and Pb(II) were rapidly adsorbed by MOMMC within 0–30 min, and the adsorption capacity and removal efficiency tended to be constant after 60 min. This indicated that a contact time of 60 min was the requirement for Cu(II) and Pb(II) to reach adsorption equilibrium on the surface of MOMMC. The adsorption kinetics data obtained experimentally were fitted to the pseudo-first-order and pseudo-second-order models. The fitting results are shown in Figure 9 and Table 4.

The pseudo-second-order model showed much better fitting to Cu(II) and Pb(II) adsorption data with higher correlation coefficients (0.9972, 0.9985) and a better agreement between experimental *Q*_e,exp_ (817.29 mg·g^−1^, 882.87 mg·g^−1^) and calculated *Q*_e,cal_ (854.70 mg·g^−1^, 909.09 mg·g^−1^). This indicated that the adsorption rates of Cu(II) and Pb(II) onto MOMMC were controlled by chemical processes [19].

### 3.5. Adsorption Isotherms of MOMMC

**Figure 10:**
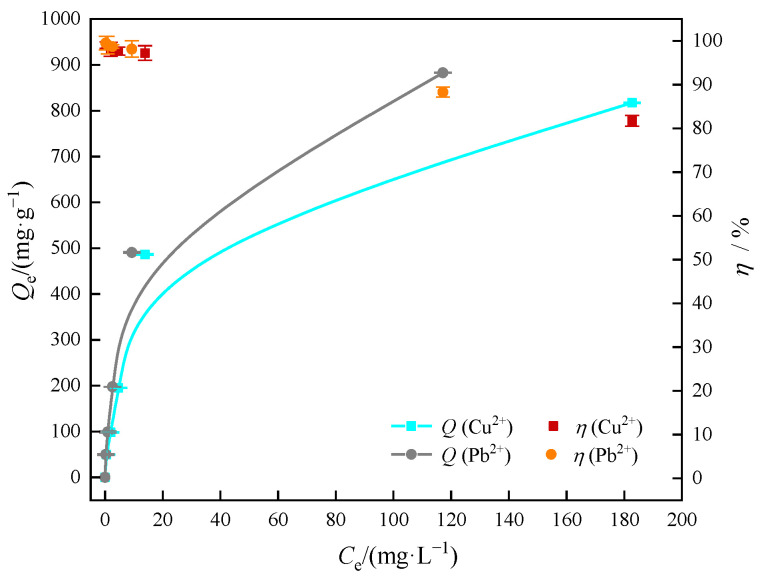
Adsorption isotherms of Cu(II) and Pb(II) on MOMMC (*C*_0_ = 1000 mg·L^−1^, pH = 5, *T* = 25 °C, adsorbent concentration = 1 g·L^−1^, contact time = 120 min).

The adsorption isotherms of Cu(II) or Pb(II) in aqueous solution by MOMMC at 25 °C are shown in Figure 10. The equilibrium adsorption capacity of Cu(II) or Pb(II) rose with the increase of the Cu(II) or Pb(II) equilibrium concentration, while the removal efficiency decreased in the meantime. This was because at a lower ion concentration, the adsorption active site of MOMMC was not fully occupied, and the increase of concentration enhanced the adsorption driving force. This trend of increase slowed down at a higher ion concentration, indicating that the unoccupied site of MOMMC was significantly reduced.

**Figure 11:**
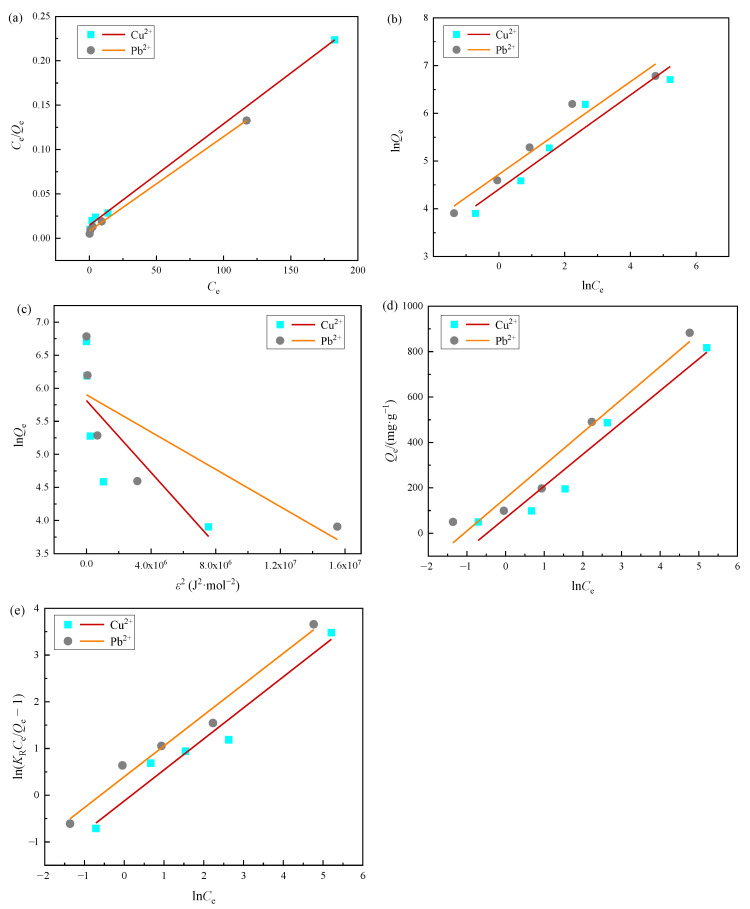
Langmuir (**a**), Freundlich (**b**), Dubinin–Radushkevich (**c**), Temkin (**d**), and Redlich–Peterson (**e**) adsorption isotherms for the adsorption of Cu(II) (**a**) or Pb(II) (**b**) on MOMMC.

Fitting curves and adsorption isotherm constants are shown in Figure 11 and Table 5. According to the highest correlation coefficients (*R*^2^ = 0.9978 for Cu(II), *R*^2^ = 0.9982 for Pb(II)) and the low average relative error (ARE = 0.1586 for Cu(II), ARE = 0.1367 for Pb(II)), the adsorption behavior of Cu(II) and Pb(II) on MOMMC was deemed to be well-described by the Langmuir model, indicating that Cu(II) and Pb(II) were mainly adsorbed by the monolayer. This might be because after the monolayer adsorption of Cu(II) or Pb(II) on MOMMC, the oxygen-containing active adsorption sites were occupied, and covalent bonds did not form anymore. According to the fitting parameters calculated from the Langmuir model in Table 4, the saturated adsorption capacity of Cu(II) at 25 °C reached 877.19 mg·g^−1^, while the saturated adsorption capacity of Pb(II) at 25 °C reached 943.40 mg·g^−1^. It is worth noting that the Redlich–Peterson model was also suitable for the adsorption of Pb(II), with a relatively high correlation coefficient (*R*^2^ = 0.9719) and a low average relative error (ARE = 0.1225). Since the Redlich–Peterson model is a combination of the Langmuir and Freundlich models [34], it is concluded that Pb(II) was mainly adsorbed by the monolayer on the surface of MOMMC, but the occurrence of multi-layer adsorption could not be ignored. This might be due to the higher electronegativity and lower hydrated radius of Pb(II) than Cu(II) [35], leading to a stronger electrostatic attraction between the surface of MOMMC and Pb(II). Multi-layer adsorption might be the reason for the higher saturated adsorption capacity of Pb(II) compared with Cu(II).

The adsorption capacity of MOMMC compared to other materials is presented in Table 6 [36,37,38,39,40,41,42].

### 3.6. Recovery Performance

**Figure 12:**
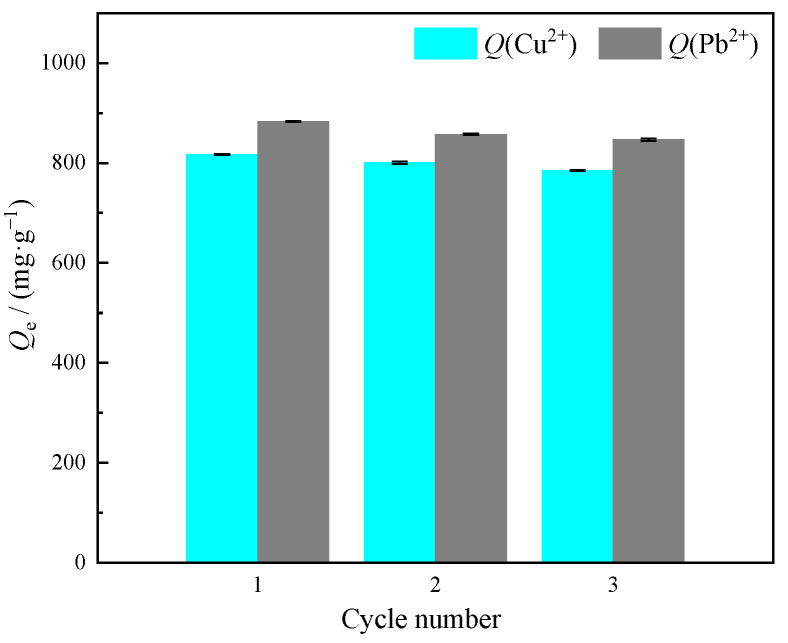
Adsorption–desorption cycles of Cu(II) and Pb(II) on MOMMC.

To gain deeper insight into the practical value, three adsorption–desorption cycles were conducted to evaluate the recyclability of MOMMC. Figure 12 represents the adsorption–desorption cycles of Cu(II) and Pb(II) on MOMMC. For the three cycles, the adsorption capacity of Cu(II) was 817.29, 800.62, and 785.16 mg·g^−1^, respectively. For Pb(II) adsorption, the adsorption capacity was 882.87, 857.44, and 846.49 mg·g^−1^. MOMMC showed stable adsorption performance to Cu(II) as well as Pb(II) after the recovery. These results meant that MOMMC was an economical adsorbent.

### 3.7. Competitive Adsorption

**Figure 13:**
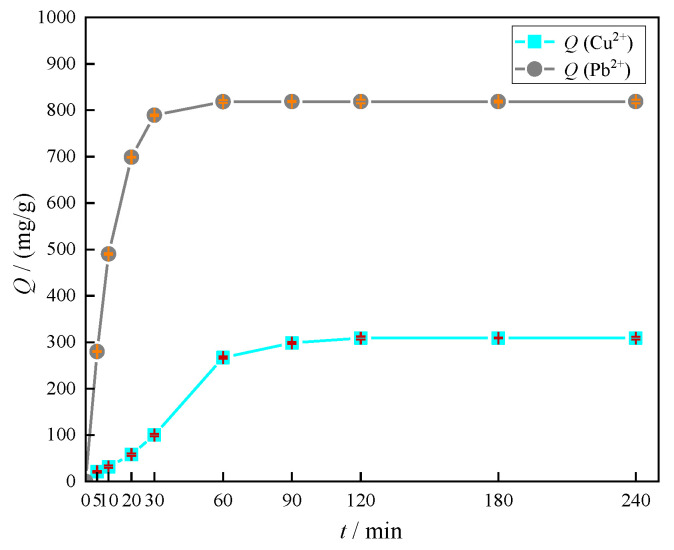
Competitive adsorption of Cu(II) and Pb(II) on MOMMC.

Competitive adsorption of Cu(II) and Pb(II) on MOMMC is shown in Figure 13. The adsorption of Pb(II) rapidly increased within 0–30 min, and tended to equilibrium after 60 min. However, for the Cu(II) adsorption, the adsorption capacity was very small within 0–30 min, increased obviously within 30–60 min, and tended to equilibrium after 120 min. This indicated that the adsorption of Cu(II) by MOMMC was inhibited by Pb(II). When Pb(II) adsorption was almost equilibrated, rapid Cu(II) adsorption started. In other words, MOMMC showed selectivity for Pb(II) adsorption, and the presence of Cu(II) had little effect on Pb(II) adsorption. This was because Pb(II) had a higher electronegativity and a lower hydrated radius, which enabled an easier approach and formation of a covalent bond with the oxygen-containing functional groups. After equilibrium, the adsorption capacity of MOMMC to Cu(II) and Pb(II) reached 309.06 and 818.83 mg·g^−1^, respectively. The removal efficiency of Cu(II) was only 30.91%, which was much lower than that of a single component, meaning that the adsorption active sites were limited and Cu(II) was mainly adsorbed by the monolayer.

### 3.8. Discussion of Mechanism

**Figure 14:**
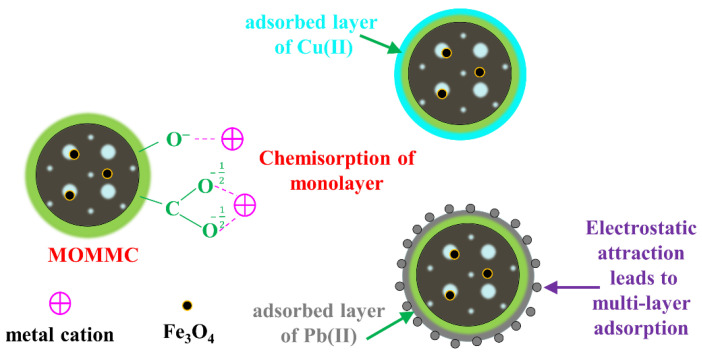
Proposed adsorption mechanism of Cu(II) and Pb(II) on MOMMC.

The possible adsorption mechanism was discussed according to the structure–activity relationship, kinetics, isotherms, and competitive adsorption. Firstly, after the oxidation and magnetization, MOMMC derived from MMC-2 showed a much smaller surface area (89.40 m^2^·g^−1^) and pore volume (0.09 cm^3^·g^−1^), but much higher adsorption capacity for Cu(II) and Pb(II). This indicated that the oxygen-containing functional groups on the surface of MOMMC played an important role in the adsorption of heavy-metal cations. A covalent bond was thought to be formed between oxygen-containing functional groups and metal cations by complexation and chelation, as well as electrostatic attraction. Secondly, kinetics studies showed that the adsorption of Cu(II) and Pb(II) on MOMMC fit the pseudo-second-order model well, indicating a rate-determining step in the chemical process. This was consistent with the conclusion of the structure–activity relationship. Thirdly, isotherm studies showed that the adsorption behavior of Cu(II) was better described by the Langmuir model, and the adsorption behavior of Pb(II) was better described by both the Langmuir and Redlich–Peterson models. This suggested that after the monolayer adsorption of Cu(II) or Pb(II) on MOMMC, the oxygen-containing active adsorption sites were occupied. However, the Redlich–Peterson model indicated that Pb(II) was adsorbed not only by the monolayer but also multi-layers on the surface of MOMMC, which was attributed to the higher electronegativity and lower hydrated radius of Pb(II) than Cu(II), leading to a stronger electrostatic attraction between the surface of MOMMC and Pb(II). Finally, the adsorption of Cu(II) by MOMMC was inhibited by Pb(II), but the presence of Cu(II) had little effect on Pb(II) adsorption. After equilibrium, the removal efficiency of Cu(II) was only 30.91%, which was much lower than that of a single component (81.73%), meaning that the adsorption active sites were limited, and Cu(II) was mainly adsorbed by the monolayer. The above four points suggested that the chemisorption caused by oxygen-containing functional groups played a major role for the adsorption of heavy-metal cations on MOMMC. A proposed adsorption mechanism of Cu(II) and Pb(II) on MOMMC is illustrated in Figure 14.

## 4. Conclusions

In this work, a magnetic oxidized micro/mesoporous carbon (MOMMC) was prepared for the easy separation of Cu(II) and Pb(II) from water, and the prepared MOMMC gained extremely high adsorption capacity for the two heavy-metal ions. All the results of the experimental studies can be summarized as follows:

(1) Micro/mesoporous carbon (MMC) was successfully prepared via the dual-template method using sucrose as the carbon source, silica nanoparticles formed by tetraethyl orthosilicate as the microporous templates, and triblock copolymer F127 as the mesoporous template. The prepared MMC gained a high specific surface area of 460.21 m^2^·g^−1^. The adsorption capacity of MMC for Cu(II) and Pb(II) reached 503.49 and 560.54 mg·g^−1^, respectively.

(2) MOMMC was obtained by oxidation using potassium persulfate and then magnetized through in situ synthesis of Fe_3_O_4_ nanoparticles. MOMMC maintained the mesoporous structure with a surface area of 89.40 m^2^·g^−1^ and pore volume of 0.09 cm^3^·g^−1^. The high-saturation magnetization (59.6 emu·g^−1^) indicated its easy and fast separation from water by an external magnetic field.

(3) Kinetics studies showed that the adsorption of Cu(II) and Pb(II) on MOMMC fit the pseudo-second-order model well, indicating a rate-determining step in the chemical process. Isotherm studies showed that the adsorption behavior of Cu(II) was better described by the Langmuir model, indicating that Cu(II) was mainly adsorbed by the monolayer. The adsorption behavior of Pb(II) was better described by both the Langmuir and Redlich–Peterson models, indicating that Pb(II) was mainly adsorbed by the monolayer on the surface of MOMMC, but the occurrence of multi-layer adsorption could not be ignored. MOMMC obtained efficient adsorption for Cu(II) and Pb(II) with the large adsorption capacity of 877.19 and 943.40 mg·g^−1^ according to the Langmuir adsorption isotherm equation.

(4) Competitive adsorption demonstrated a better selectivity for Pb(II) on MOMMC, and MOMMC still possessed a large adsorption capacity for Cu(II) and Pb(II) after three adsorption–desorption cycles. The chemisorption caused by oxygen-containing functional groups played a major role for the adsorption of heavy-metal cations on MOMMC.

Further research will be conducted on the adsorption of various pollutants, including heavy-metal ions, organic pollutants, and biomacromolecules. In addition, various modifications will be considered, such as amination and sulfhydrylation.

## Figures and Tables

**Table 1:** Detailed recipes for the prepared micro/mesoporous carbon samples.

Sample	F127/g	Hydrochloric Acid/mL	Ethanol/mL	Sucrose/g	TEOS/g
MMC-1	1.80	0.5	5.0	0.90	0.37
MMC-2	1.80	1.0	10.0	1.80	0.73
MMC-3	1.80	3.0	30.0	5.40	2.19

**Table 2:** Physical quantities in five adsorption isotherm models.

Physical Quantity	Abbreviation	Values and Units
Equilibrium adsorption capacity	*Q*_e_	mg·g^−1^
Equilibrium concentration	*C*_e_	mg·L^−1^
Maximum adsorption capacity	*Q*_m_	mg·g^−1^
Langmuir adsorption isotherm constant	*K*_L_	L·mg^−1^
Freundlich adsorption isotherm constant	*K*_f_	(mg·g^−1^) (L·mg^−1^)^1/n^)
Heterogeneity parameter	*n*	/
Polanyi potential	*ε*	*ε* = *RT*ln(1 + 1/*C*_e_), J·mol^−1^
Constant related to adsorption-free energy	*β*	mol^2^·J^−2^
Gas constant	*R*	8.3145 J·mol^−1^·K^−1^
Absolute temperature	*T*	K
Temkin isotherm constant	*b*_T_	J·mol^−1^
Temkin isotherm equilibrium constant	*A*_T_	L·g^−1^
Redlich–Peterson constants	*K*_R_	L·g^−1^
Redlich–Peterson constants	*g*	/
Redlich–Peterson constants	*B*	L·g^−1^
Experimental equilibrium adsorption capacity	*Q*_e,exp_	mg·g^−1^
Calculated equilibrium adsorption capacity	*Q*_e,cal_	mg·g^−1^

**Table 3:** Pore structure parameters of MMC-1, MMC-2, MMC-3, and MOMMC.

Sample	Specific Surface Area/(m^2^·g^−1^)	Specific Surface Area of Micropore/(m^2^·g^−1^)	Total Pore Volume/(cm^3^·g^−1^)	Micropore Volume/(cm^3^·g^−1^)	Mesopore Volume/(cm^3^·g^−1^)	Total Pore Size/nm	Mesopore Size/nm	Type	Hysteresis
MMC-1	183.53	/	0.41	/	0.42	7.50	8.21	IV	H2(b)
MMC-2	460.21	170.24	0.82	0.06	0.76	7.12	8.19	IV	H2(b)
MMC-3	381.72	290.12	0.26	0.12	0.14	3.43	8.14	I + IV	H4
MOMMC	89.40	26.64	0.09	0.01	0.08	3.37	5.18	IV	H2(b)

**Table 4:** Fitting parameters of adsorption kinetics for Cu(II) and Pb(II) adsorption on MOMMC.

Systems	*Q*_e,exp_ /mg·g^−1^	Pseudo-First-Order			Pseudo-Second-Order		
		*Q*_e,cal_ /mg·g^−1^	*k*_1_ /min^−1^	*R* ^2^	*Q*_e,cal_ /mg·g^−1^	*k*_2_ /g·mg^−1^·min^−1^	*R* ^2^
**Cu(II)**	817.29	167.47	0.062	0.8172	854.70	0.00014	0.9972
**Pb(II)**	882.87	160.60	0.061	0.8472	909.09	0.00020	0.9985

**Table 5:** Adsorption isotherm parameters and error analysis for the adsorption of Cu(II) or Pb(II) on MOMMC.

Models.	Parameters	Cu(II)	Pb(II)
Langmuir	*Q*_m_/mg·g^−1^	877.19	943.40
	*K*_L_/L·mg^−1^	0.0783	0.1299
	*R* ^2^	0.9978	0.9982
	ARE	0.1586	0.1367
Freundlich	*n*	2.02	2.06
	*K*_f_/((mg·g^−1^) (L·mg^−1^)^1/n^)	82.1864	112.1851
	*R* ^2^	0.9069	0.9355
	ARE	0.2278	0.1970
Dubinin–Radushkevich	*Q*_m_/mg·g^−1^	334.73	365.45
	*β*/mol^2^·J^−2^	2.72×10^−7^	1.41×10^−7^
	*R* ^2^	0.4697	0.5278
	ARE	11.4106	14.2571
Temkin	*b*_T_/J·mol^−1^	17.6809	17.1163
	*A*_T_/L·g^−1^	1.6204	2.9214
	*R* ^2^	0.9314	0.9412
	ARE	0.5730	0.5771
Redlich–Peterson	*g*	0.6645	0.6605
	*K*_R_/L·g^−1^	150	300
	*B*/L·g^−1^	0.8846	1.4860
	*R* ^2^	0.9473	0.9719
	ARE	0.1549	0.1225

**Table 6:** Comparison of adsorption capacity.

Adsorbent Material	Cu(II)/mg·g^−1^	Pb(II)/mg·g^−1^	References
CTS cross-linked with CA-ECH	151.52	121.95	[36]
γ-Fe_2_O_3_@HAp	88.2		[37]
SA-PVA-fungus GXCR bead	1.107	1.618	[38]
SA-PVA-GO-fungus GXCR bead	1.71	1.589	[38]
SA-PVA-AC-fungus GXCR bead	1.551	1.638	[38]
Fe_3_O_4_@SiO_2_@mC	48.5	94.0	[39]
Fe_3_O_4_@SiO_2_@mC-H_2_O_2_	86.5	156	[39]
Ordered mesoporous carbon		288.4	[40]
Boron-doped mesoporous carbon		415.5	[40]
Ordered mesoporous carbons containing amino propyls	4.97	6.86	[41]
Mesoporous activated carbon from kenaf	12.7	12.0	[42]
Magnetic oxidized micro/mesoporous carbon	877.19	943.40	This study

## Data Availability

Data presented in this study are available upon request from the first author.
